# The Relations of the Tooth-Pulp to the Other Tooth Tissues

**Published:** 1890-04

**Authors:** George H. McCausey

**Affiliations:** Janesville, Wis.


### International Dental Journal.

VOL. XI. APRIL, 1890. No. 4.

### Original Communications.[1]

### THE RELATIONS OF THE TOOTH-PULP TO THE OTHER TOOTH TISSUES.[2]

BY GEORGE H. M'CAUSEY, JANESVILLE, WIS.

OUR imagination is never at rest. During the hours of sleeping it may run riot. During the hours of waking it may be controlled or modified by judgment.

The judgment, controlling the imagination, may have been perfected through a knowledge of facts, and the extent of that knowledge will determine to how great an extent the imagination may be controlled by judgment.

If we neglect the means of acquiring knowledge, we neglect the very means of perfecting our judgment, and we may find that which we consider reason to be merely a grade of imagination.

The human animal acquires knowledge through the medium of the five organs of sense, either of which is created for its own special function. Certain varieties of wine, for instance, can hardly be distinguished apart when presented to the sight, but experts readily determine a variety by placing it in contact with the taste-buds of the tongue.

[1] The editor and publishers are not responsible for the views of authors of papers published in this department, nor for any claim to novelty, or otherwise, that may be made by them. No papers will be received for this department that have appeared in any other journal published in this country.

[2] Read before the New York Odontological Society, January 21, 1890.

The subtile perfume floating in the atmosphere can be smelled, but who ever felt or saw it? Yet the use of more than one sense may be necessary, at times, to determine certain truths. The interpretation placed upon the impressions conveyed to the brain, through either one or all of the human senses, may and does often differ, yet that does not excuse a refusal to employ the senses in the effort to arrive at or approximate truth.

The unassisted organs of sense are, however, limited in the extent to which they may serve us, and the inventive genius of mankind is being continually exerted in an effort to aid them in the performance of their function. As the Creator has created in the human organism certain organs with special functions, so the necessities of the human family have led to the invention of mechanism for special uses. The necessities of the astronomer led to the invention of the telescope, without the aid of which his occupation would be gone; and instead of a knowledge of facts, so far as facts can be determined through its use, he would be engaged in speculations, which amount simply to Scotch verdicts, or, in other words, not proven. If astronomical facts are arrived at through the use of the telescope, a theory formulated by an astronomer who will use it may approximate the truth. But what shall we say of a theory founded on nothing, which, through the aid of the telescope, has been demonstrated as a fact? If he will use it, he may learn much; if not, can he learn anything definite? If the principle is true, in case of the astronomer, it is no less so in case of the histologist.

There is a limit to the function of the eye, and beyond which the sight cannot penetrate, unless aided by the microscope, which to the eye of the histologist is an annex through which he arrives at truths, which can be arrived at by no other means at present known; and it is therefore safe to assume that any histological theory may be questioned, if not based upon facts ascertained to be such through its use; and any histological theory which cannot at present be demonstrated through its use may be considered as not proven, and as yet an open question.

In a consideration of the relations of the tooth-pulp to the other tooth tissues, the writer assumes that the microscope is the only medium through which we can arrive at facts regarding that important organ, and while awaiting the result of the future effort of the makers of microscope objectives, we can only pursue our studies by aid of the best productions of the present time, and which, like the unaided eye, have a limit to their performance.

A knowledge of the relations of the tooth-pulp to the other tooth tissues involves a study of the developing tooth, and is a question of histology and function.

Sections of teeth and jaw of the lower animals, at about the time of birth, enable us to study the relations of the tooth-pulp to the other tooth tissues sufficiently near, to determine regarding the commencement of the relation, and which can afterwards be pursued by sections yet older, and up to the time of full development. It is a recognized fact that all tissues to be examined under the microscope should be subjected as little as possible to the action of any agent which has a tendency to shrivel the elements, and, in the treatment of embryonal tissues particularly, it should be avoided. Therefore such sections should never be mounted in balsam, which involves the use of alcohol for dehydration. The method described by Dr. Andrews, of Cambridge, Mass., has been found by the writer best to preserve the structure in its original form as a permanent mount. Sections are examined best while resting in a medium which possesses as nearly as possible the same refractive power as do the fluids of the body, and the writer has found iodized amniotic liquor to answer the purpose admirably. The sections may be examined under objectives ranging from a one-eighth upward. They should be of high angle, and, if possible, either oil or homogeneous immersion.

Objections have been raised against the use of the term "tooth-pulp," but, in the opinion of the writer, it is better to use a term which, while it falls short of conveying a full idea of structure and function, is not positively misleading as to facts; and until some better term can, in accordance with a knowledge of facts, be devised, it is, in my opinion, better to retain and for the time make use of the term tooth-pulp. At the instant of the commencement of proliferation of the bud of embryonal subepithelial tissue (and which becomes the future organ of dentinal formation) there begins a series of changes which are to determine the relations of the tooth-pulp to the other tooth tissues.

The growth of the bud continues in an upward direction against the epithelial elements of the enamel organ, until, through the resistant pressure of the enamel organ, it becomes nearly enclosed by the enamel organ. At this particular epoch we can commence advantageously a study of that tissue which constitutes the dental pulp. If we focus upon the tissue (prepared as before recommended) an objective of first class definition and sufficient power of amplification we shall find the central portion to be composed of the simplest kind of tissue, that known as mucous, or myxomatous. Certain of the cells will be found to contain homogeneous protoplasm only. Others show finely granular bodies within the protoplasm, while others, in addition to the granules, exhibit nuclei. Other cells can be found of the form which we know as bipolar, and are those from which arise the connective-tissue fibres which are found embedded in the pulp at its maturity.

At its periphery we find yet other forms of protoplasmic bodies, and which stand like columns, perpendicular to its surface. One form at its outward extremity is found to be square, or nearly so. Others are of a pear-shaped form, the smaller extremity pointing outward. Both forms will at their outward extremities be found to show processes which, apparently, are a continuation of the protoplasmic contents of the cells of which they are processes. At this time there will be found surrounding the tissue, which we have examined, a layer of formed dentine, and into which we readily trace the processes of the peripheral cells of the pulp.

The fact that the peripheral cells form the only portion of tissue in immediate contact with the forming dentine, justly leads to the conclusion that these cells are the only medium through which the cartilaginous matrix of the dentine is formed and its subsequent calcification is effected.

Observation of sections yet older in development shows that increase in thickness of the dentine is accompanied by a corresponding decrease in the diameter of the pulp.

If we next examine our first section of embryonal pulp tissue under a higher power we will find the cell walls to be penetrated by most delicate filaments, which are connected with the granules of the protoplasm of the cells, the granules themselves being connected with each other by the same delicate filaments. These filaments which penetrate the cell wall are continued through the walls of the adjoining cells, thus forming a delicate net-work throughout the tissue of the entire pulp, including its columnar and pear-shaped cells. Blood-vessels will be found penetrating the tissue of the pulp, the significance of which, as in all tissues, is that of nutrition.

The blood furnished through the developed and developing vessels nourishes the tissues of the pulp, at the same time furnishing the pabulum from which is elaborated the materials for the structure of the forming dentine, through the medium of the peripheral cells.

As all mature tissue has arisen primarily from the mucoid tissue of the embryo, so does the dentine arise through the formative action of the pulp which, having performed its function, yet continues as an organ of repair, when necessity calls for such action. Unlike most tissues, the thoroughly developed pulp consists largely of its embryonal elements, the only change being in the elements contained within it. Thus far no allusion has been made to the nerves of the pulp, but which we will now consider as we see them through the microscope objective.

We will try and ascertain if we have the right to call a part the whole or the whole a part. An old theory—which is so very old that to most of us it has become new—is that the pulp of a tooth is a nerve. It is an idea which, in the dim past, was communicated by the physician to the patient, and from parent to child among the laity, and it is through the admirable performance of the modern objective that we are to prove the theory a truth or a fallacy.

Later on, we have been told that the function of the pulp is that of a ganglion or nerve-centre. If the tooth-pulp is a nerve we shall expect to find it to be composed wholly of the elements which enter into the structure of nerve tissue. If it be a nerve, it must be morphologically of one or another of the forms of neural tissue. If its function is that of conduction of nervous influence, we will find it composed entirely of nerve fibres. But we have already found it to be composed of mucoid tissue. Microscopical examination reveals the fact that it, like other tissues, contains within its substance neural tissue of that form known as tubular neurine, which enters into the structure of nerves whose function is the conduction of nervous influence.

If the pulp is a ganglion, we may expect to find it consisting of neural tissue in the form of cells.

But as mucoid tissue cannot consist of other elements than mucoid elements, and at the same time retain its identity as mucoid tissue, we will not expect to find it consisting of vesicular neurine, as elements entering into the structure of ganglia. Being formed by the union of nerves, ganglia may be expected to be found connected with axis cylinders, as poles of their cells. We find nothing of the kind, however, unless, by a long stretch of the imagination, we assume that the processes of the peripheral cells are nerve terminals. That assumption is very hard to prove, as their behavior, under the action of reagents and stains, is very different from that which we know to be nerve tissue, when submitted to the same treatment.

Instead of the tooth-pulp showing any of the characteristics of a ganglion, or other nerve tissue, we find that, like other tissues, it contains neural tissue within itself, and of that form which the histologist recognizes as medullated.

The fibres traverse the structure of the pulp mostly in a direction parallel with its long axis, but, at times, approach its periphery, where they terminate as exceedingly small non-medullated fibrils, which may rest near to or between the formative cells of the dentine. That, during the excavation of a cavity of decay in dentine, contact of the instrument with the processes of the formative cells produces a sense of pain in greater or less degree, every dentist well knows; but that it is caused by direct contact of the instrument with nerve tissue, the writer is not at the present time prepared to admit. He has been quoted as denying that the dentinal tubules contain nerve tissue. That assertion is a mistake, but he takes the present opportunity to say that he does not believe that they contain nerve tissue, but is open to conviction.

At the present time it is with him an open question, and the only theory which he has to advance to account for the existence of pain in excavation is through the contractility of the protoplasmic processes of the peripheral cells of the pulp, and the impression of which may be received by the nerve terminals through the medium of the protoplasmic net-work, which extends through and connects the cellular elements of the entire pulp; and it is with him simply a theory which he has as yet been unable to demonstrate to his entire satisfaction as the truth. If we admit that the tooth-pulp is either histologically or functionally a ganglion, we must, in order to be consistent, admit that each and every papilla of the corium which contains nerve terminals is a ganglion, the fact that one is surrounded by a wall of calcified tissue, and the other not so surrounded, having no particular significance. If we admit that the tooth-pulp is a ganglion, we seek in vain for the organ which forms the cartilaginous matrix of the dentine, and afterwards plays an important part in its calcification, and we are compelled to admit, at the same time, that the science of histology is a rope of sand and the results of the continued studies and patient plodding of Virchow, Bilroth, Stricker, Schultze, Heitzman, Black, *et al.*, have gone for naught. The patient investigations of Legros and Magitot have no practical significance, so far as their conclusions are concerned. We are forced to the conclusion that antiseptic treatment of a tooth containing a dead "ganglion" is a myth, and the almost marvellous performance of objectives made by Spencer,

Bausch and Lomb, Tolles, Zeiss, *et al.*, representing the result of the highest grade of patience and skill, absolutely wasted. The years of toiling by skilful manipulators of such wonderful lenses have been worse than thrown away. But the honest seeker after truth may take renewed courage in the thought that, the theory that the tooth-pulp is a ganglion, from either a histological or functional stand-point, would never have been formulated by even a mediocre manipulator of the microscope.

